# Changes in Ovulation-Related Gene Expression during Induced Ovulation in the Amur Sturgeon (*Acipenser schrenckii*) Ovarian Follicles

**DOI:** 10.3390/ijms232113143

**Published:** 2022-10-29

**Authors:** Ryohei Surugaya, Yuya Hasegawa, Shinji Adachi, Shigeho Ijiri

**Affiliations:** Graduate School of Fisheries Sciences, Hokkaido University, Hakodate 041-8611, Hokkaido, Japan

**Keywords:** luteinizing hormone, maturation-inducing steroids, ovulatory competence, sturgeon, oocyte maturation, ovulation, teleosts, ovulation-related gene

## Abstract

The luteinizing hormone (LH) and maturation-inducing steroids (MIS), such as 17α,20β-dihydroxy-4-pregnen-3-one, regulate the final oocyte maturation in teleosts. Oocyte maturational competence (OMC) and ovulatory competence measure the sensitivity to MIS for oocyte maturation and ovulation, respectively. However, the molecular mechanisms underlying the acquisition of ovulatory competence remain unknown. Sturgeons are an excellent research model for investigating these mechanisms. We examined the seasonal profiles of OMC and ovulatory competence in vitro and the expression of 17 ovulation-related gene candidates using quantitative PCR in Amur sturgeon ovarian follicles. The ovulatory competence was induced by the LH-releasing hormone analog (LHRHa) priming injection after acquiring the OMC, which was spontaneously induced in spring or autumn. Seven genes, including the tissue-type plasminogen activator (*plat*), were enhanced following the LHRHa priming injection in ovarian follicles sampled from anovulated and ovulated fish. The activin receptor type 1 (*acvr1*) and prostaglandin G/H synthase 2 (*ptgs2*) were only upregulated in ovulated fish. Our results suggest that *plat*/plasmin and prostaglandin (PG)/PG receptor systems are essential for sturgeon ovulation, similar to other vertebrates. Notably, successful ovulation depends on a sufficient PG synthesis, and mediators activating the PG/PG receptor system are essential for acquiring the ovulatory competence. We provide the first report of ovulation-related gene alterations in the ovarian follicles of Amur sturgeons.

## 1. Introduction

The final oocyte maturation in teleosts is typically regulated by the luteinizing hormone (LH), gonadotropin secreted from the pituitary, and maturation-inducing steroids (MIS). When the oocyte completes vitellogenesis, the massive secretion of LH (LH surge) induces the follicular cell layer to produce MIS, consequently inducing a germinal vesicle breakdown (GVBD)—the marker of oocyte maturation—with meiotic resumption and ovulation. In amago salmon (*Oncorhynchus masou ishikawae*), 17α,20β-dihydroxy-4-pregnen-3-one (DHP) was identified as an MIS for the first time in vertebrates [1]. In the Atlantic croaker (*Micropogonias undulatus*), 17,20β,21-trihidroxy-4-pregnen-3-one (20β-S) was identified as an MIS [2]. Since then, DHP and 20β-S have been identified as an MIS in many teleosts. It has recently been shown that DHP is highly likely to be an MIS in sturgeons (Hasegawa et al., unpublished data). In addition, oocytes may acquire an oocyte maturational competence (OMC) in a process where oocytes acquire a sensitivity to MIS before meiosis is restarted by an MIS stimulation [3]. In vitro, OMC can be determined by a GVBD induction in response to MIS. OMC is induced via an LH stimulation in kisu (*Sillago japonica*), tobinumeri-dragonet (*Repomucenus beniteguri*), and zebrafish (*Danio rerio*) [4,5,6]. OMC in red seabream (*Pagrus major*) is similarly acquired in response to the LH and not by the follicle-stimulating hormone [7]. OMC in ayu (*Plecoglossus altivelis*) is triggered by an LH-induced increase in cyclic adenosine monophosphate (cAMP) concentration in the follicular cells and the subsequent activation of the protein kinase A system by the flow of cAMP into the oocyte through the gap junctions between the follicular cells and the oocytes themselves [8].

If the oocyte has an OMC, oocyte maturation and ovulation should occur smoothly once it has been stimulated with MIS. In contrast, in some teleosts, such as the northern pike (*Esox lucius*), rainbow trout (*Oncorhynchus mykiss*), and African clawed toad (*Xenopus laevis*), GVBD is induced but a subsequent ovulation may not be induced even in the presence of MIS [9,10,11]. Therefore, the ability of ovarian follicles to ovulate once exposed to MIS, called “ovulatory competence,” must be acquired before ovulation. Ovulatory competence is likely induced via an LH stimulation after the OMC is acquired in the Atlantic croaker, Japanese eel (*Anguilla japonica*), and hybrid bester sturgeon (*Huso huso* × *Acipenser ruthenus*) [12,13]. However, little is known about the ovulatory competence, and the detailed molecular mechanisms underlying the acquisition of the ovulatory competence remain unknown in all species.

Ovarian follicles that have acquired an ovulatory competence are stimulated by MIS to ovulate. Ovulation is thought to occur through two processes: hydrolysis of the extracellular matrix (ECM) of the follicular cell layer (degradation of the follicular wall) and follicle contraction. Proteases have long been proposed to cause ovulation by lysing the follicle wall [14], and plasmin and collagenase have attracted attention as enzymes that induce this process. Plasmin is produced by a plasminogen activator (PA), and PA activity has been shown to increase with ovulation in rats (*Rattus norvegicus*) and cattle (*Bos taurus*) [15]. Plasmin degrades the basement membranes in medaka (*Oryzias latipes*) [16,17]. Several matrix metalloproteinases (MMPs) with a collagenase activity have been identified as ovulatory enzymes in medaka [18]. MMPs are candidate enzymes for lysing the follicular wall in many vertebrates [19].

Prostaglandins (PGs) have also long been known to induce ovulation [20,21]. PGs were first shown to be present in human semen in 1936 and have been reported to have hypotensive and smooth muscle-contractive actions [22,23]. In rainbow trout, PGF_2α_ induces ovulation via a muscle-like contraction of the theca cell layer [24]. In the medaka, PGE_2_ induces ovulation by binding to the PGE_2_ receptor [25]. Based on this knowledge from vertebrates, PA/plasmin and MMP systems are largely responsible for the degradation of the follicular wall, and the PG/PG receptor system is largely responsible for the follicular wall contraction. However, the molecular mechanisms of the follicular wall lysis and contraction have only been investigated in detail in the medaka and remain to be elucidated in many teleosts.

Acipenseriformes (25 species of sturgeon and two paddlefish) are experiencing a dramatic decline in their population worldwide due to environmental degradation and overfishing. All species are listed as endangered on the IUCN Red List (https://www.iucnredlist.org/ accessed on 27 August 2022). Since 1988, all sturgeon species have been regulated by the Convention on International Trade in Endangered Species of Wild Fauna and Flora. However, the ovaries of these species are still in a high demand owing to their high economic value as caviar. Therefore, establishing a stable seedling production technology to produce caviar without natural resources and restore those resources is desirable. In Acipenseriformes, the complete vitellogenesis of oocytes occurs in captivity; however, oocyte maturation does not occur spontaneously. Oocyte maturation and ovulation are currently stimulated by the administration of hormones, such as carp or sturgeon pituitary extract [26,27], the LH-releasing hormone (LHRH), or its analog LHRHa [28,29]. However, ovulation may not be induced even though oocyte maturation is induced [30,31] because ovarian follicles did not acquire an ovulatory competence before an LHRHa injection. Therefore, to accurately predict the timing of ovulation induction, elucidating the ovulation mechanism, particularly the molecular mechanism of an ovulatory competence induction, is crucial.

In teleosts, gene analysis has not been conducted using the samples collected during the process of acquiring an ovulatory competence. This is because observing this phenomenon in fish model organisms, such as medaka and zebrafish, which have short spawning cycles, is challenging. Meanwhile, sturgeons in captivity have a long spawning cycle of 3–4 years [29]. The OMC and ovulatory competence induction takes several months [32], allowing for a detailed analysis of the molecular mechanism involved in acquiring ovulatory competence. Using ovarian follicles from sterlets (*Acipenser ruthenus*), we have previously identified several candidates for ovulation-related genes whose mRNA levels increased before or after the acquisition of ovulatory competence using the RPKM index with RNA-sequencing analysis (unpublished data).

In this study, we investigated the seasonal changes in the OMC, ovulatory competence, and mRNA levels of ovulation-related genes in the Amur sturgeon (*Acipenser schrenckii*). In addition, we compared the expression of these ovulation-related genes between the fish that reached ovulation and those that did not to infer the process of acquiring an ovulatory competence. Thus, we aimed to provide a foundation for further research on sustainable sturgeon breeding practices to promote seedling production.

## 2. Results

### 2.1. Seasonal Changes of OMC and Ovulatory Competence in Ovarian Follicles

**Figure 1:**
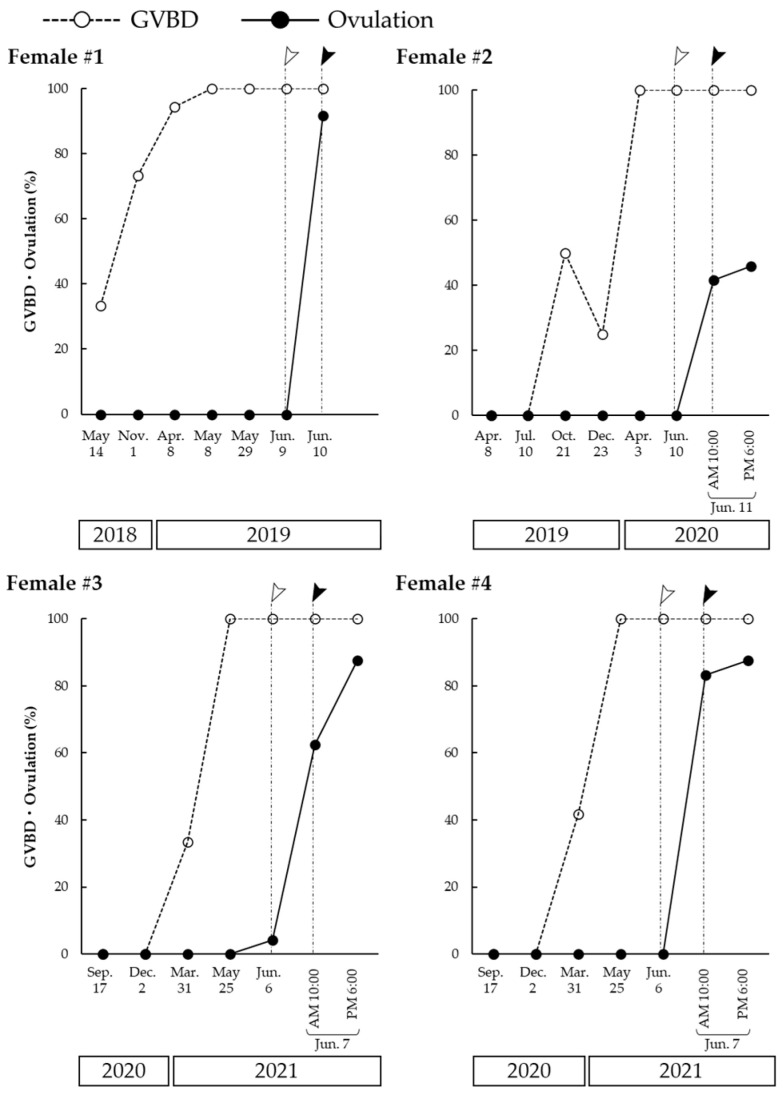
Seasonal changes in oocyte maturational and ovulatory competence in females #1–4. White and black arrowheads indicate the date of luteinizing hormone-releasing hormone analog (LHRHa) priming injection and high-dose injection administration, respectively. Ovarian follicles were biopsied just before each injection. Follicles were incubated with 100 ng/mL 17α-hydroxyprogesterone (17OHP), and germinal vesicle breakdown (GVBD)/ovulation rates were examined.

In vivo, ovulation was induced approximately 24 h after an LHRHa high-dose injection in females #1–4. The ovarian follicles of four Amur sturgeons were cultured for 48 h in the presence of 17OHP to confirm that the follicles possess the OMC and ovulatory competence (Figure 1). We used 17OHP as a precursor of MIS, and it has a maturation-inducing activity comparable with MIS [33]. Follicles incubated without 17OHP as a control did not induce an oocyte maturation or ovulation in any fish at any sampling point except for 24 h after the priming injection and 8 h after the high-dose injection. The GVBD and ovulation in response to 17OHP are the markers of OMC and ovulatory competence, respectively.

In females #1 and #2, most oocytes achieved an OMC in April. In females #3 and #4, approximately one-third of the oocytes acquired an OMC by the end of March, and all the oocytes acquired an OMC in May. Nevertheless, the number of oocytes that acquired an OMC was 100% in the spring before the LHRHa priming injection. In females #1, #3, and #4, most of the ovarian follicles acquired an ovulatory competence 24 h after the priming injection, and approximately half of the ovarian follicles in female #2 also acquired an ovulatory competence. Furthermore, in female #3, a few follicles had an ovulatory competence just before the priming injection.

### 2.2. Seasonal Changes of Ovulation-Related Gene mRNA Expression in Ovarian Follicles

**Figure 2:**
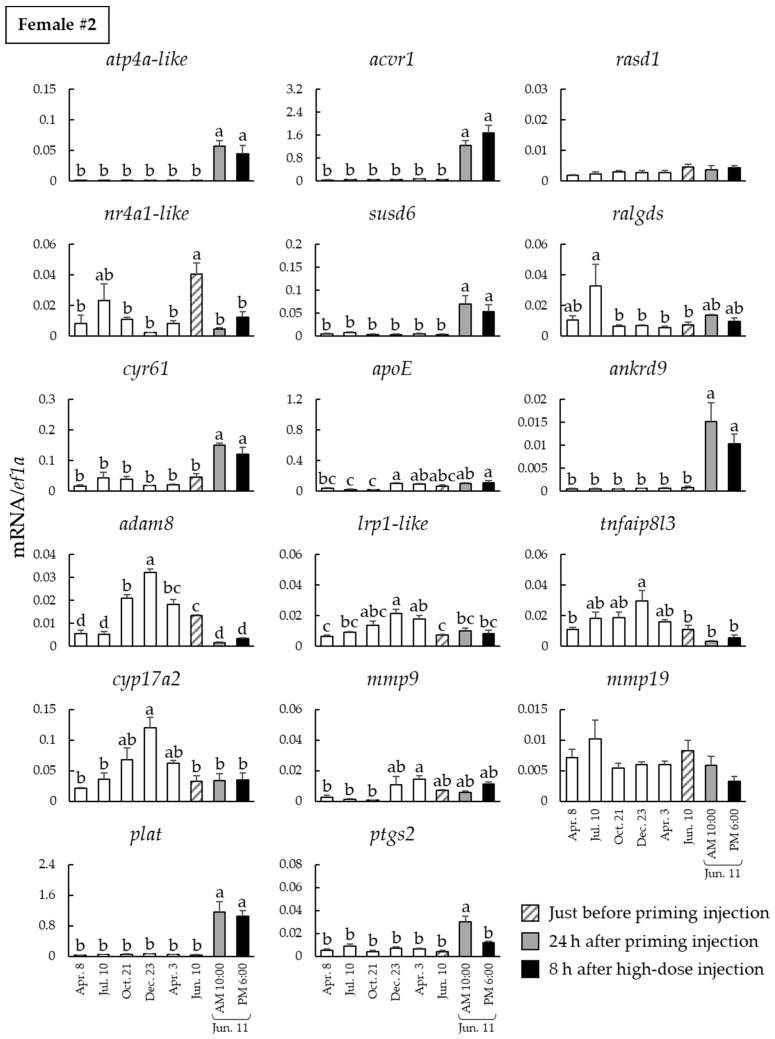
Seasonal changes of ovulation-related gene mRNA expression in the ovarian follicles of female #2. Expression levels of three follicles isolated on 8 April 2019, to 3 April 2020 (open column), just before LHRHa priming injection (hatched column), 24 h after priming injection (shaded column), and 8 h after high-dose injection (solid column) were administered. Data are presented as the mean values ± standard error of the mean (SEM), and different letters indicate significant differences set at *p* < 0.05.

We examined the seasonal changes in the mRNA levels of the ovulation-related genes in the ovarian follicles of females #1–4 (Figure 2, Supplemental Figures S2–S4). The mRNA levels of potassium-transporting ATPase alpha chain 1-like (*atp4a-like*), activin receptor type 1 (*acvr1*), sushi domain-containing protein 6 (*susd6*), cysteine-rich angiogenic inducer 61 (*cyr61*), ankyrin repeat domain-containing protein 9 (*ankrd9*), tissue-type plasminogen activator (*plat*), and prostaglandin G/H synthase *2* (*ptgs2*) were significantly increased after the priming injection in all fish. In addition, the mRNA levels of the ras dexamethasone-induced protein 1 (*rasd1*) and apolipoprotein E (*apoE*) were significantly increased after the priming injection in all fish, except for female #2. In contrast, the mRNA levels of disintegrin and metalloprotease domain 8 (*adam8*) were significantly lower after the priming injection in all fish, except for female #4.

### 2.3. PI in Oocytes of Anovulated and Ovulated Fish

**Figure 3:**
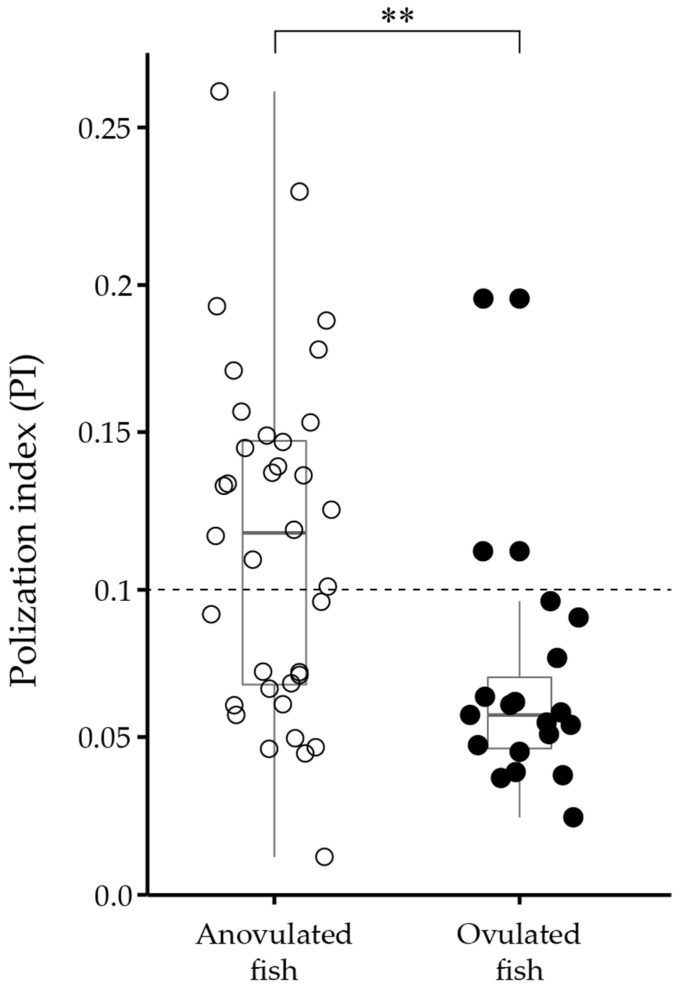
Polarization index (PI) of oocytes isolated from 36 anovulated (open circle) and 19 ovulated fish (solid circle) just before priming injection was administered. Asterisks indicate a significant difference set at *p* < 0.01 (**) (*t*-test).

The ovarian follicles were collected from the Amur sturgeons immediately before the priming injection, and the PI of the oocytes was calculated (Figure 3). The PI of the oocytes from ovulated fish was less than 0.1, except for two sturgeons. The average PI of the oocytes from anovulated fish was 0.116, and the smallest and largest PI were 0.0125 and 0.2636, respectively.

### 2.4. Expression Changes of Ovulation-Related Genes in Anovulated and Ovulated Fish

**Figure 4:**
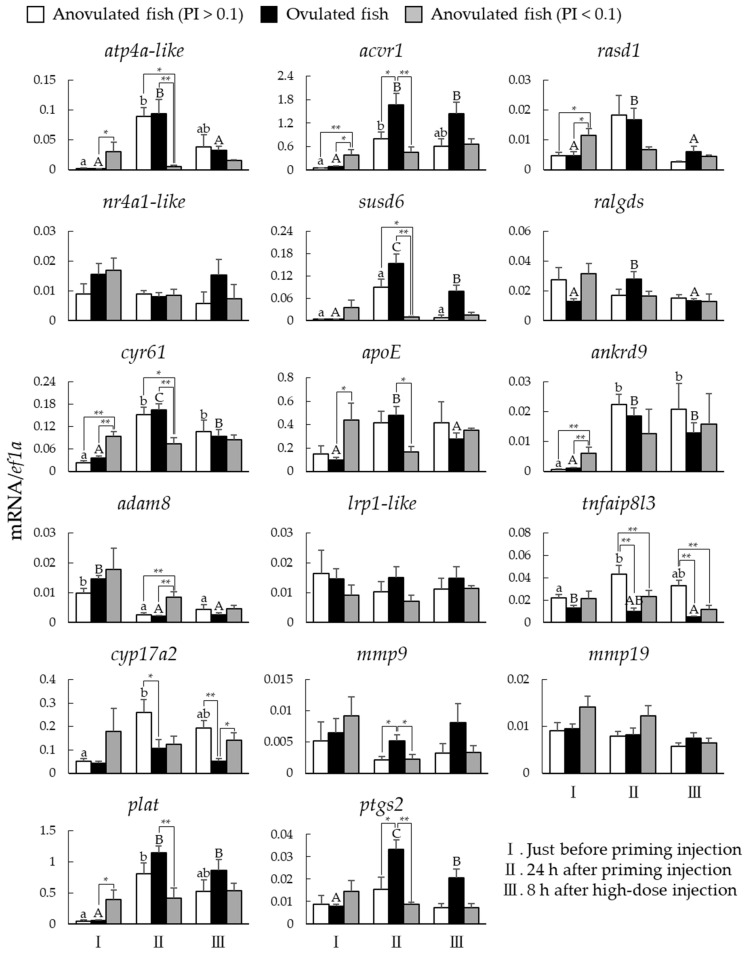
mRNA levels of ovulation-related genes in ovarian follicles of anovulated and ovulated fish. Expression levels in anovulated (PI > 0.1; open column), ovulated (solid column), and anovulated fish (PI < 0.1; shaded column). Follicles isolated just before priming injection (I), 24 h after priming injection (II), and 8 h after high-dose injection (III) were administered. Data are presented as the mean values ± SEM (n = 3–10) and different letters (Anovulated fish (PI > 0.1), lowercase letters; ovulated fish, uppercase letters) indicate significant differences set at *p* < 0.05. * *p* < 0.05, ** *p* < 0.01.

We measured the mRNA levels of the ovulation-related genes in the ovarian follicles of anovulated (PI > 0.1 or PI < 0.1) and ovulated fish using qPCR and compared their expression before and after the LHRHa injection (Figure 4). Information on the Amur sturgeons’ samples is presented in Table 1. The mRNA levels of *atp4a-like*, *rasd1*, *susd6*, *cyr61*, *apoE*, *ankrd9*, and *plat* were higher in anovulated (PI > 0.1) and ovulated fish after the priming injection. In contrast, the mRNA levels of *acvr1* and *ptgs2* were increased by the priming injection only in ovulated fish. The mRNA levels of *adam8* tended to decrease after the priming injection in all fish. The mRNA levels of the tumor necrosis factor alpha-induced protein 8-like protein 3 and cytochrome P450 family 17 subfamily A polypeptide 2 increased by the priming injection only in anovulated fish (PI > 0.1). In anovulated fish (PI < 0.1), the mRNA levels of *atp4a-like*, *rasd1*, *susd6*, *cyr61*, *apoE*, *ankrd9*, *plat, acvr1*, and *ptgs2* were not remarkably induced after the priming injection, but tended to be slightly higher just before the priming injection than in anovulated (PI > 0.1) or ovulated fish.

## 3. Discussion

In this study, we investigated the seasonal profiles of the OMC and ovulatory competence in Amur sturgeons. The results revealed that the OMC induction began in autumn through to the spring and it was completed in the spring. In contrast, the ovulatory competence was scarcely acquired by the seasonal changes but was induced predominantly with a priming injection.

The pattern of the LH surge varies among the teleosts, with a relatively gradual increase in the serum LH levels in salmonids [34]. In goldfish (*Carassius auratus*) and carp (*Cyprinus carpio*), the LH was slightly secreted prior to the LH surge, and the serum LH levels rapidly increased several hours before ovulation [35,36,37]. A gradual increase in the LH prior to its surge was expected to induce an ovulatory competence. The priming injections of low concentrations of LH increased the ovulation rates in rainbow trout and Japanese eels [10,38]. This may be because a priming injection mimics the gradual increase in the LH and promotes the acquisition of ovulatory competence. In bester sturgeons, OMC is triggered at least three months before the induction of ovulatory competence, after which the ovulatory competence is spontaneously acquired in the autumn or the following spring [32]. The timing of the acquisition of the OMC and ovulatory competence differs from that of besters, which may be due to the nervousness of Amur sturgeon. In domestic gilthead seabreams (*Sparus aurata*) and striped basses (*Morone saxatilis*), some females were found to progress to oogenesis without a hormonal treatment [39]. A similar phenomenon has been observed in white sturgeon (*Acipenser transmontanus*) aquacultured traditionally, where a small percentage of oocytes spontaneously complete a GVBD without a hormone administration [40]. In Amur sturgeons, which have been artificially bred for only a few generations, it was considered that ovulatory competence was scarcely triggered without an exogenous treatment because of non-domestication.

We also investigated the changes in the expression of the ovulation-related genes using the ovarian follicles from the Amur sturgeons. *atp4a-like*, *acvr1*, *rasd1*, *susd6*, *cyr61*, *apoE*, *ankrd9*, *plat*, and *ptgs2* appear to be LH-inducible genes. A recent study on oocyte maturation in Amur sturgeon reported that the serum DHP concentration and mRNA levels of *hsd17b12L*, which is an enzyme that converts 17OHP to DHP, increased 24 h after the LHRHa priming injection [41]. In addition, the oocytes of females #1–4 completed the GVBD 24 h after the priming injection in vivo (Supplemental Figure S5); therefore, these nine genes may be expressed after the ovarian follicles acquire a complete ovulatory competence.

In contrast, matrix metalloproteinase 9 (*mmp9*) and matrix metalloproteinase 19 (*mmp19*) were not induced following the LHRHa priming injection. MMPs, a family of zinc-dependent endopeptidases, are usually produced as zymogens and activated by the removal of the propeptide regions. MMPs have been reported to be involved in ovulation in a variety of organisms [42,43,44]; some are LH-inducible MMPs, whereas others are not [18,45,46]. In this study, the transcriptional levels of *mmp9* and *mmp19* did not clarify whether they are involved in ovulation in Amur sturgeons.

A disintegrin and metalloproteinase (ADAM) family and a disintegrin and metalloproteinase with thrombospondin motifs (ADAMTS) family, which are closely related to the MMP gene family, have also been reported to be involved in ovulation in vertebrates [47]. In mice, *adam8* expression is induced in granulosa and cumulus cells by the combination of the LH and nuclear progestin receptor (nPR or Pgr) in preovulatory follicles [48]. *adam8b* expression in the follicular cell layer increased rapidly just before ovulation in wild-type zebrafish, whereas its expression diminished in the *nPR*-knockout fish [43]. In two sterlets used in the experiment, *adam8* was induced during the incubation with SPE (Supplemental Figure S6). In the Amur sturgeon, *adam8* reached a maximal expression a few months before the priming injection was administered, indicating that it may be induced by low concentrations of LH. Thus, *adam8* may be promoted by the LH-induced nPR in Amur sturgeons; however, the timing of the *adam8* expression in Amur sturgeons appears to be different from that of sterlets. The *adam8* in Amur sturgeons droped sharply after the priming injection was administrated, probably because the *adam8* had completed its role. These changes in the nPR expression necessitate further study. However, *adam8* is not expected to be a determinant of a successful ovulation induction since the peak of its expression was also present in individuals who did not reach ovulation.

Considering the combination of the PI index of oocytes sampled before the priming injection from ovulated and anovulated fish, most ovulated fish had a PI of less than 0.1, whereas that of anovulated fish ranged from 0.012 to 0.263. The degree of GV migration is an indicator of oocyte maturity, and the closer the GV is to the animal pole, the greater the sensitivity of the oocyte to MIS [49]. In white sturgeons, oocytes with a PI < 0.125 had a 100% OMC [40]. PI is often used as an index to judge the timing of ovulation induction in sturgeons, and Chapman and Van Eenennaam suggested that hormone injections lead to a successful ovulation induction if the fish show a PI < 0.1 [50]. However, a large number of sturgeons in this study failed to ovulate despite having oocytes with a PI < 0.1. Oocytes can wait until the onset of maturation after the completion of vitellogenesis; however, if this time is too long, they become overripe in the follicle and then undergo follicular atresia. Sturgeons and Japanese eels frequently have overripe preovulatory ovarian follicles in their ovaries, and delayed ovulation or anovulation often occurs [51]. Therefore, oocytes with a PI < 0.1 are highly likely to experience preovulatory overripeness in the Amur sturgeon, whereas oocytes with a PI > 0.1 are immature.

We measured the mRNA levels of the ovulation-related genes in the ovarian follicles sampled from anovulated (PI > 0.1 or PI < 0.1) and ovulated fish before and after an LHRHa injection. The mRNA levels of *atp4a-like*, *susd6*, *cyr61*, *ankrd9*, and *plat* increased following the LHRHa priming injection in both anovulated (PI > 0.1) and ovulated sturgeons. Significant upregulation of *apoE* and *rasd1* followed the LHRHa priming injection only in the ovulated sturgeons, but there was no significant difference between the anovulated (PI > 0.1) and ovulated fish at 24 h after the LHRHa priming injection. These results suggest that these genes were prematurely ready to be induced well before the LHRHa priming injection.

*rasd1* is an activator of G protein signaling and a monomeric G protein that belongs to the RAS superfamily of GTPases. Recently, *rasd1* knockout mice do not undergo a normal oocyte maturation, suggesting that *rasd1* is an important factor that regulates cytokinesis, spindle formation, and signal transduction during the mid-meiosis I to mid-meiosis Ⅱ transition (MI/MⅡ oocyte transition) [52].

*cyr61*, a member of the CCN family, is known to negatively regulate collagen homeostasis, and in primary human skin dermal fibroblasts, *mmp1* expression is synchronized with that of *cyr61* [53]. In mice, a treatment with ulipristal acetate, a progesterone receptor ligand that inhibits ovulation but not cumulus expansion or follicle growth, resulted in lower levels of *cyr61* mRNA compared to that in the controls. The expression of *cyr61* is downregulated in nPR knockout mice [54]. Perhaps, *cyr61* may regulate the collagen degradation through the transcriptional promotion of MMPs downstream of nPR in the follicular cell layer of sturgeons.

*plat* converts plasminogen to active plasmin, which is a serine proteinase. The plat/plasmin system is involved in ovulation. In rats and cattle, the activity of plat in the follicle wall fraction was shown to synchronously increase with the LH levels toward ovulation [15,55]. Furthermore, tranexamic acid, which inhibits PA synthesis, suppresses ovulation in golden hamsters (*Mesocricetus auratus*) [56]. There are two types of PAs: tissue and urokinase. Urokinase-type PA, urokinase-type plasminogen activator 1, is responsible for the plasminogen activation during medaka ovulation, and then the produced plasmin degrades the laminin in the basement membrane [16,17]. Thus, PA/plasmin-mediated follicular wall lysis is a common mechanism in vertebrates, and the plat/plasmin system is expected to be important for ECM remodeling during ovulation in sturgeons.

Notably, the *acvr1* and *ptgs2* expression was induced only in the ovulated sturgeons. Mediators of the expression of these genes may be prepared in the late stages of ovulatory competence induction.

*acvr1* is a receptor for the bone morphogenetic protein (BMP), and its expression has been confirmed in bovine and equine ovarian follicles. In cattle, the *acvr1* mRNA levels in granulosa cells tended to increase with an increasing follicle size [57]. In mares, the protein levels were reported to be approximately three times higher in the granulosa cells of preovulatory follicles than in mid-ovulatory follicles without the mRNA expression levels [58], suggesting the importance of the activation of the BMP signaling via Acvr for ovulation. Culturing cumulus oophorus complexes from pregnant horse serum gonadotropin (PMSG)-treated mice in the presence of BMP15 increased the *ptgs2* expression, which is involved in prostaglandin synthesis [59]. Additionally, activin A, via *acvr1b*, upregulated the *ptgs2* expression in human granulosa-lutein cells [60].

*ptgs2* is a PG synthase. Various PGs are synthesized from arachidonic acid via a common intermediate, PGH2, and *ptgs2* metabolizes arachidonic acid to PGH2. In zebrafish, *ptgs2a* is induced by LH signaling in an *npr*-independent manner just before ovulation after the GVBD in the follicular cell layer, and ovulation is suppressed in the ovarian follicles incubated with indomethacin, a ptgs inhibitor [61,62]. The LH stimulates the *ptgs2* expression in the brown trout (*Salmo trutta*), African clawed toad (*Xenopus laevis*), and mammals, such as humans, mice, and rhesus monkeys (*Macaca mulatta*) [63,64,65,66,67]. In addition, Jalabert et al. suggested that GVBD, but not ovulation, is induced by the lack of mediators that initiate the contraction of the follicular wall [10]. Similarly, the qPCR analyses implied that the inability to induce a follicular wall contraction due to the insufficient expression of *ptgs2* may be connected to ovulatory dysfunction in sturgeons. In fact, when immature ovarian follicles were cultured in vitro in the presence of 17OHP, partially exposed through the openings in the follicular layers, although they did not completely ovulate (Supplemental Figure S7). Follicle wall lysis, including the degradation of the basement membrane by plasmin, had likely progressed to a certain extent, even in immature ovarian follicles.

In anovulated fish (PI < 0.1), the mRNA levels of several ovulation-related genes were shown to be significantly high just before the LHRHa priming injection, suggesting that they might be markers for intrafollicular overripeness. In African clawed toads with intrafollicular-overripe follicles, the non-disjunction of the chromosomes in the oocytes have been suggested to bring about the delay of ovulation [68]. In addition, when hCG was administered to rats 12 h after the estimated time of ovulation, it was observed that many large ovarian follicles of ovulatory size hardly reached ovulation [69]. In Amur sturgeons with ovarian follicles that were intrafollicular overripe, some ovulation-related genes may not have been induced for transcription by the LHRHa injection because of the loss of the LH sensitivity (e.g., inadequate LH release due to captivity).

**Figure 5:**
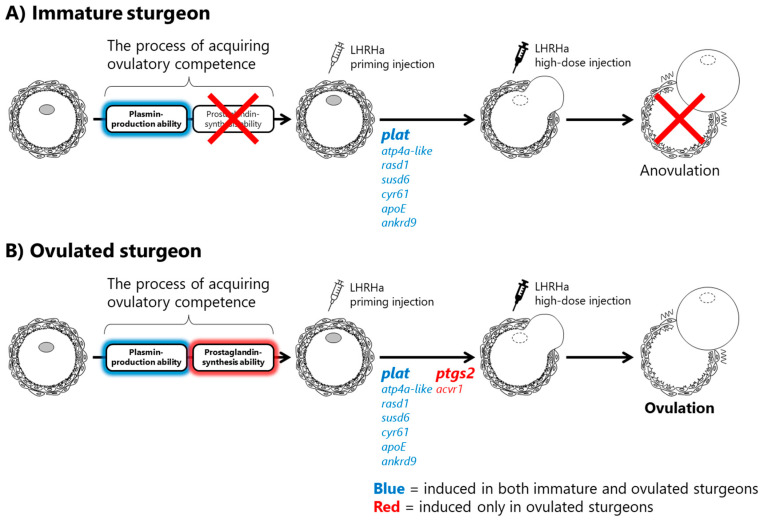
Estimated ovulation process in the Amur sturgeon. (**A**) In immature sturgeons, only plasmin-producing ability is acquired before the priming injection and then 7 ovulation-related genes (blue) were expressed after the priming injection. (**B**) In ovulated sturgeons, genes shown in red are induced after the priming injection, in addition to the genes shown in blue, because their ovarian follicles have the ability to synthesize prostaglandins even before the priming injection.

In summary, changes in the expression of the ovulation-related genes in the ovarian follicles of Amur sturgeons were clarified for the first time, suggesting that plat/plasmin and PG/PG receptor systems are important for ovulation in sturgeons, similar to other vertebrates. Figure 5 shows an outline of the ovulation process predicted in this study. (A) In immature sturgeons, the competence of plasmin production was acquired, and *plat* was induced by the LHRHa priming injection. (B) The competence of PG synthesis, in addition to the plasmin production, was acquired before the LHRHa priming injection was administered in ovulated sturgeons. Thus, priming injections can enhance the mRNA levels of *ptgs2* and *acvr1*. In other words, the plasmin-producing ability is acquired much earlier in the process of an ovulatory competence acquisition, whereas the PG-producing ability is acquired at the end of the process, and successful ovulation depends on a sufficient PG synthesis. The preparation of mediators related to the activation of the PG/PG receptor system is probably essential for the completion of the ovulatory competence acquisition.

## 4. Materials and Methods

### 4.1. Experimental Fish and Sample Collection

In this study, 55 adult female Amur sturgeons were used. Sturgeons were raised outdoors in river or spring water under natural day length at Nanae Fresh-Water Station (Nanae, Hokkaido, Japan), Bifuka Sturgeon Museum (Bifuka, Hokkaido, Japan), or Shimizugawa Trout Farm (Hachimantai, Iwate, Japan). Final oocyte maturation and ovulation were induced by an LHRHa (Sigma-Aldrich, St. Louis, MO, USA) priming injection (2 μg/kg body weight) and a high-dose LHRHa injection (50 μg/kg body weight), 24 h after the priming injection.

Ovarian fragments were obtained by biopsy from females #1–26 anesthetized with approximately 0.01% of 2-phenoxyethanol (FUJIFILM Wako Pure Chemical Corporation, Osaka, Japan) immediately before the priming injection, 24 h after the priming injection, and 8 h after the high-dose injection (Table 1). In addition to the above sampling schedule, females #1–4 were routinely biopsied the year prior to the administration of the priming injection. The samples were stored in Ringer’s solution modified for the sturgeons (111.2 mM NaCl, 3.4 mM KCl, 2.7 mM CaCl_2_, and 23.8 mM NaHCO_3_; pH 7.4) at 4 °C until the start of the experiment.

### 4.2. In Vitro Culture of Ovarian Follicles

The separated ovarian follicles of females #1–4 were incubated in individual wells of a 24-well culture (AGC TECHNO GLASS Co., Ltd., Shizuoka, Japan) in Leibovitz’s L-15 medium (Sigma-Aldrich) containing 0.062 g/L of penicillin, 0.1 g/L of streptomycin, 0.05 g/L of kanamycin, and 2.52 g/L of EPPS and the pH was modified to 8.2 with NaOH. To investigate the maturity state in detail, 24 ovarian follicles were incubated in 1 mL of Leibovitz’s L-15 medium with or without 100 ng/mL of 17α-hydroxyprogesterone (17OHP) for 48 h at 12 °C. The oocyte maturation and ovulation rates were calculated after 48 h of incubation using the same evaluation criteria as Ishihara et al. in 2014 [29].

### 4.3. Calculation of Polarization Index (PI)

The ovarian follicles which were biopsied immediately before the priming injection in 55 females, including females #1–26, were fixed with Bouin’s solution. These fixed samples were strongly dehydrated using ethanol and butanol and then embedded in paraffin. The samples were sliced into 5–10 µm thick sections and stained with hematoxylin and eosin. To calculate the PI of the oocytes, the diameter without the oocyte membrane along the animal-vegetal axis (A) and the distance between the top edge of the germinal vesicle (GV) and the oocyte membrane (B) of the tissue sections were measured using ImageJ (https://imagej.nih.gov/ij/, accessed on 8 May 2018) (Supplemental Figure S1). The PI was calculated using the following formula:

PI = B/A

The average of 10 oocytes was considered per sturgeon.

### 4.4. RNA Extraction and Quantitative PCR (qPCR)

The ovarian follicles biopsied from females #1–26 were stored in RNA*later* stabilization solution (Thermo Fisher Scientific Inc., Waltham, MA, USA) at −30 ℃ until the RNA extraction. The total RNA was isolated from three yolkless follicles from each sturgeon using ISOGEN reagent (Nippon Gene Co., Ltd., Toyama, Japan). The cDNA was reverse-transcribed from 700 ng of total RNA using ReverTra Ace reverse transcriptase (Toyobo Co., Ltd., Osaka, Japan) and random hexamer primers (Thermo Fisher Scientific Inc.) in 15 μL reactions. Then, the cDNA was diluted 100-fold, and 2 μL was used in a 10 μL qPCR reaction. The mRNA levels of the 17 ovulation-related genes (primers are listed in Table 2) were measured by qPCR using a PowerUp SYBR Green Master Mix (Thermo Fisher Scientific Inc.) and the StepOnePlus Real-Time PCR System (Thermo Fisher Scientific Inc.). The mRNA levels of these genes were normalized against those of the housekeeping gene, elongation factor 1-alpha.

### 4.5. Statistical Analysis

A statistical analysis was performed using Excel Statistical Analysis Ver. 7.0 (ESUMI Co. Ltd., Tokyo, Japan). The mRNA levels of the ovulation-related genes were compared between groups using the Tukey–Kramer test. The data are expressed as the mean ± standard error of the mean (SEM), and significant differences were set at *p* < 0.05. The PI was compared between the ovulated and anovulated fish using the *t*-test, and the significant differences were set at *p* < 0.01.

## Figures and Tables

**Table 1:** PI and sampling term in 26 Amur sturgeons.

Sample No.	PI	Ovulation	Sampling Term *^1^
#1	0.038	Success	I–II *^2^
#2	0.060	Success	I–III *^2^
#3	0.025	Success	I–III *^2^
#4	0.049	Success	I–III *^2^
#5	0.053	Success	I–III
#6	0.063	Success	I–III
#7	0.040	Success	I–III
#8	0.056	Success	I–III
#9	0.039	Success	I–III
#10	0.047	Success	I–II
#11	0.135	Failure	I–III
#12	0.110	Failure	I–III
#13	0.134	Failure	I–III
#14	0.138	Failure	I–II
#15	0.126	Failure	I–II
#16	0.137	Failure	I–II
#17	0.110	Failure	I–II
#18	0.131	Failure	I–II
#19	0.092	Failure	I–III
#20	0.069	Failure	I–III
#21	0.059	Failure	I–III
#22	0.051	Failure	I–III
#23	0.062	Failure	I–II
#24	0.048	Failure	I–II
#25	0.012	Failure	I–II
#26	0.046	Failure	I–II

*^1^ The sampling time was just before priming injection (I), 24 h after priming injection (II), and 8 h after high-dose injection (III) were administered. *^2^ Females #1–4 were routinely biopsied in the year prior to the priming injection besides I–III.

**Table 2:** Gene name and primer sequences for qPCR.

Gene Name	Symbol	Primer Name	Primer Sequence
Potassium-transporting ATPase alpha chain 1-like	*atp4a-like*	*atp4a-like*-F	5′-GACTGGAGACCAAGAACATCGC-3′
		*atp4a-like*-R	5′-GATGGCGATGGGAGTCTTCTC-3′
Activin receptor type 1	*acvr1*	*acvr1*-F	5′-GTTCTGCGCCGTGTGG-3′
		*acvr1*-R	5′-GCTGTGAATGGCTCTGTCC-3′
Ras dexamethasone-induced protein 1	*rasd1*	*rasd*-F	5′-CAAGGATGACGATGCGTATGG-3′
		*rasd*-R	5′-CATCAAGTCACTGTGGACACTCG-3′
Nuclear receptor subfamily 4 group A member 1	*nr4a1-like*	*nr4a1-like*-F	5′-GAGGACGCCAGCGACATC-3′
		*nr4a1-like*-R	5′-GATGTTCACTGCCCACTTGC-3′
Sushi domain-containing protein 6	*susd6*	*susd6*-F	5′-GGAGTTCATAGCACCTTACCG-3′
		*susd6*-R	5′-CAGGACAGAGCCCAGACAG-3′
Ral guanine nucleotide dissociation stimulator	*ralgds*	*ralgds*-F	5′-GCACAGCGATAGAGGGAGAG-3′
		*ralgds*-R	5′-CGAACTGTCCTCATTGAACGC-3′
Cysteine-rich angiogenic inducer 61	*cyr61*	*cyr61*-F	5′-GAAAGCCAGAAGTGCATCG-3′
		*cyr61*-R	5′-CTGGTGCATTTCTTTCCCTTC-3′
Apolipoprotein E	*apoE*	*apoE*-F	5′-CGAGAAGTTGGAACCCTACACC-3′
		*apoE*-R	5′-CTTGAACTCCTCTGCCTTGCTG-3′
Ankyrin repeat domain-containing protein 9	*ankrd9*	*ankrd9*-F	5′-GACCGGCACGACTACATG-3′
		*ankrd9*-R	5′-AGCAACAGAACCAAACACTC-3′
A disintegrin and metalloprotease domain 8	*adam8*	*adam8*-F	5′-TGTGGAAAGATACATTGTCAGG-3′
		*adam8*-R	5′-GACATCTCTGCTATTTTACACTGC-3′
Low density lipoprotein receptor-related protein 1-like	*lrp1-like*	*lrp1-like*-F	5′-TGGGTGGACGCTTACTACGAC-3′
		*lrp1-like*-R	5′-GCCAAAGGCGTGGTTCAG-3′
Tumor necrosis factor alpha-induced protein 8-like protein 3	*tnfaip8l3*	*tnfaip8l3*-F	5′-TGAATCAGAGCGCCATG-3′
		*tnfaip8l3*-R	5′-CGTTGAGGAGTTCGGAGAG-3′
Cytochrome P450 family 17 subfamily A polypeptide 2	*cyp17a2*	*cyp17a2*-F	5′-CAGGCTGGGGTGCAAGTATG-3′
		*cyp17a2*-R	5′-GCAATGTCTTTCCCATCCCTG-3′
Matrix metalloproteinase 9	*mmp9*	*mmp9*-F	5′-CTTTGACACCGACAAGAAATACG-3′
		*mmp9*-R	5′-CAATCACTGCTGTGTCTCGG-3′
Matrix metalloproteinase 19	*mmp19*	*mmp19*-F	5′-CTCAGTCCTGTCTGCGGTGC-3′
		*mmp19*-R	5′-CTGAGCGCCTCCTCGATC-3′
Tissue-type plasminogen activator	*plat*	*plat*-F	5′-GAACCCAGACAACGACTCCAAG-3′
		*plat*-R	5′-CTTGGGCAGGTCGCAGTG-3′
Prostaglandin G/H synthase 2	*ptgs2*	*ptgs2*-F	5′-AGACCCTTGACAGGCAGCA-3′
		*ptgs2*-R	5′-GTACATCTCCCCATCAATCATCTG-3′
Elongation factor 1 alpha	*ef1a*	*ef1a*-F	5′-TGGCATGATCGTCACCTTTG-3′
		*ef1a*-R	5′-GCATTTCCACAGACTTCACTTCAG-3′

## Data Availability

Not applicable.

## Supplementary Materials

The supporting information can be downloaded at: https://www.mdpi.com/article/10.3390/ijms232113143/s1.
