# Supplementary material for: Changes in Ovulation-Related Gene Expression during Induced Ovulation in the Amur Sturgeon (Acipenser schrenckii) Ovarian Follicles
Source: Int J Mol Sci. 2022 Oct 29;23(21):13143. doi: 10.3390/ijms232113143 (PMC9656806; doi:10.3390/ijms232113143)
Supplement: Supplementary file 1 [file ijms-23-13143-s001.zip › ijms-1956031-supplementary.pdf]

Supplementary Figure Legends

Supplemental Figure S1. Tissue section of an Amur sturgeon ovarian follicle stained with hematoxylin and eosin. To calculate the polarization index (PI) of oocytes, the diameter without oocyte membrane along the animal-vegetal axis (A) and the distance between the top edge of the germinal vesicle (GV) and the oocyte membrane (B) were measured in the tissue sections. PI was calculated using the following formula: PI = B/A. Scale bar = 1 mm.

Supplemental Figure S2. Seasonal changes in ovulation-related gene mRNA expression in female #1 ovarian follicles. Expression levels of three follicles isolated on May 14, 2018, to May 29, 2019 (open column), just before luteinizing hormone-releasing hormone analog (LHRHa) priming injection (hatched column), and 24 h after priming injection (shaded column) was administered. Data are presented as mean ± SEM, and different letters indicate significant differences set at $P < 0.05$.

Supplemental Figure S3. Seasonal changes in ovulation-related gene mRNA expression in female #3 ovarian follicles. Expression levels of three follicles isolated on Sep. 17, 2020, to May 25, 2021 (open column), just before LHRHa priming injection (hatched column), 24 h after priming injection (shaded column), and 8 h after high-dose injection (solid column) were administered. Data are presented as mean ± SEM, and different letters indicate significant differences set at $P < 0.05$.

Supplemental Figure S4. Seasonal changes in ovulation-related gene mRNA expression in female #4 ovarian follicles. Expression levels of three follicles isolated on Sep. 17, 2020, to May 25, 2021 (open column), just before LHRHa priming injection (hatched column), 24 h after priming injection (shaded column), and 8 h after high-dose injection (solid column) were delivered. Data are presented as mean ± SEM, and different letters indicate significant differences at $P < 0.05$.

Supplemental Figure S5. Changes in germinal vesicle breakdown (GVBD) rates in vivo following LHRHa injection in females #1–4. White and black arrowheads indicate the date of the LHRHa priming injection and high-dose injection administration, respectively. Ovarian follicles were biopsied immediately prior to each injection.

Supplemental Figure S6. *Adam8* mRNA levels in ovarian follicles from two sterlets during incubation with salmon pituitary extract (SPE). The results for control and SPE are shown in grey and blue column, respectively. Data are presented as mean ± SEM, and different letters indicate significant differences at $P < 0.05$. *$P < 0.05$, **$P < 0.01$, ***$P < 0.001$.

Supplemental Figure S7. Immature ovarian follicles that stopped in the middle of ovulation. The rupture site in the follicle cells is indicated by a white arrowhead.

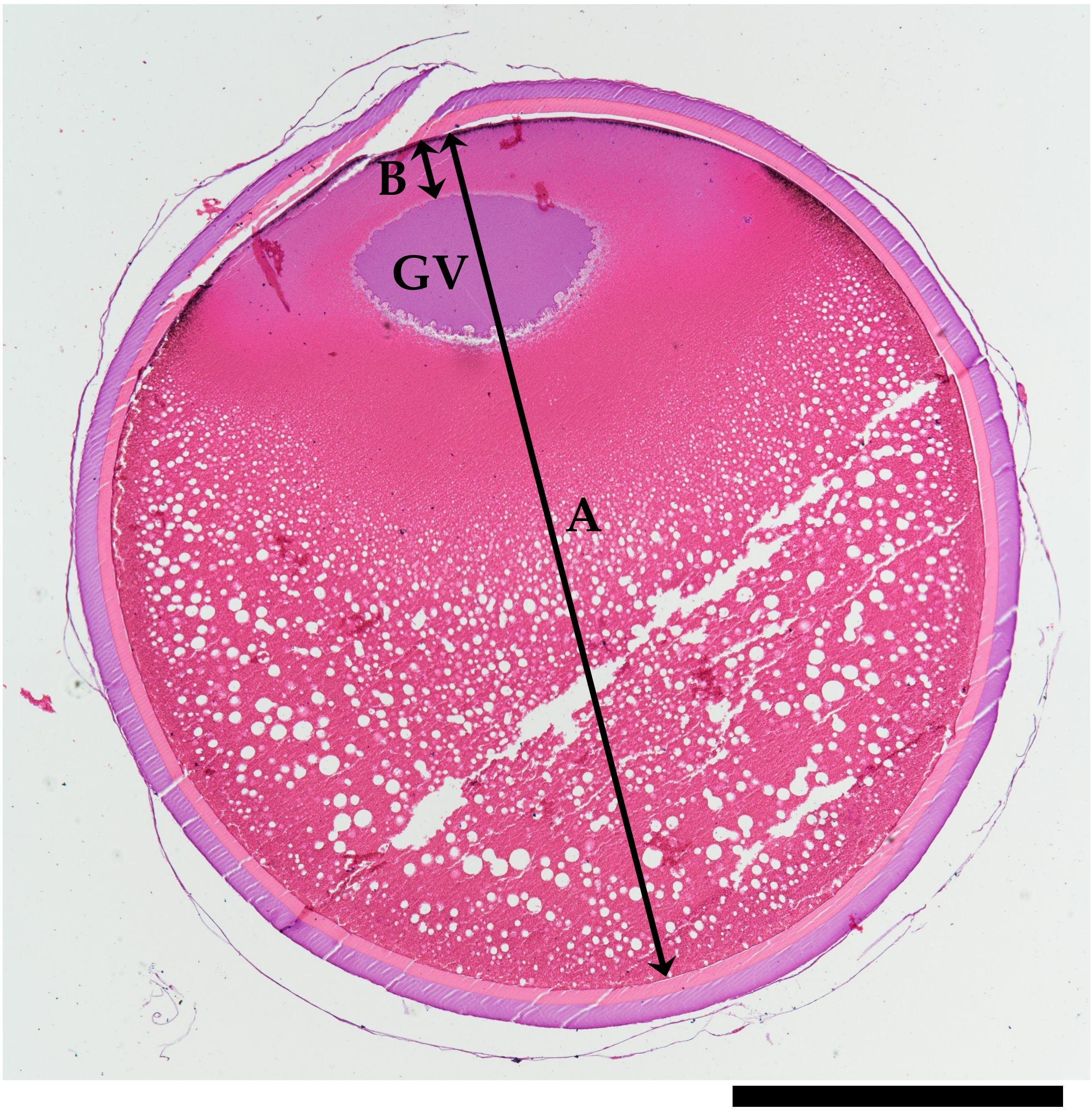

Supplemental Figure S1

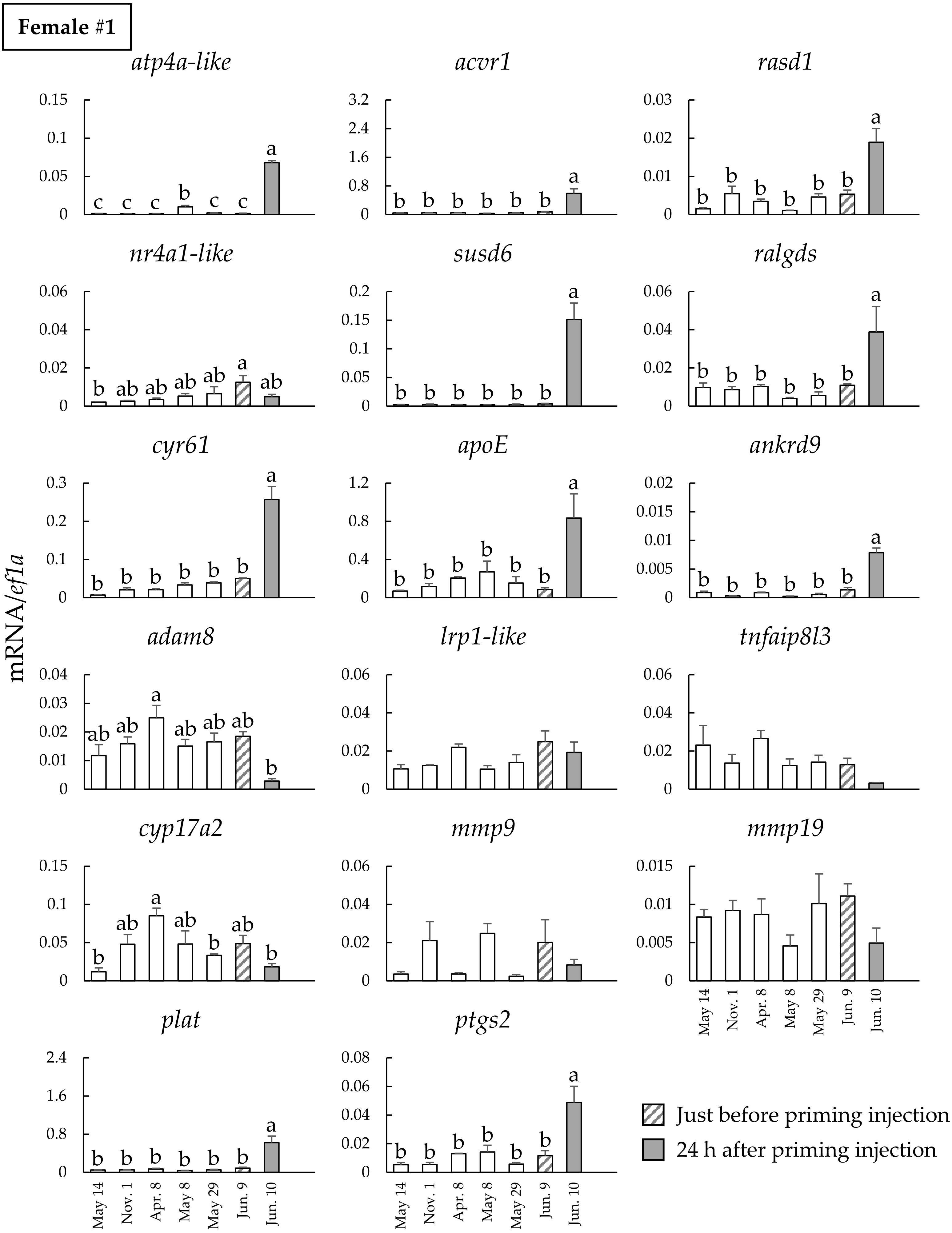

Supplemental Figure S2

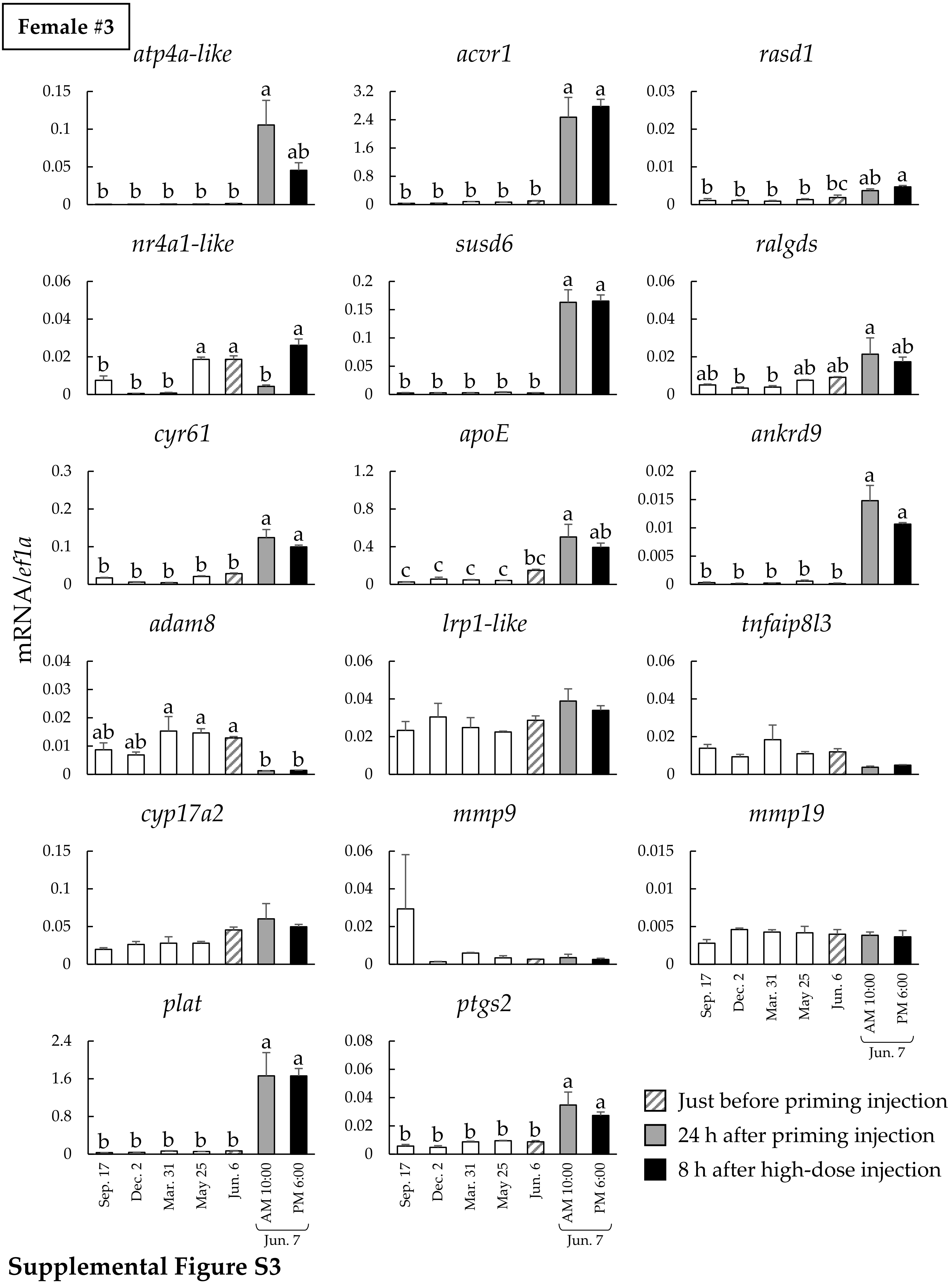

Supplemental Figure S3

Female #4

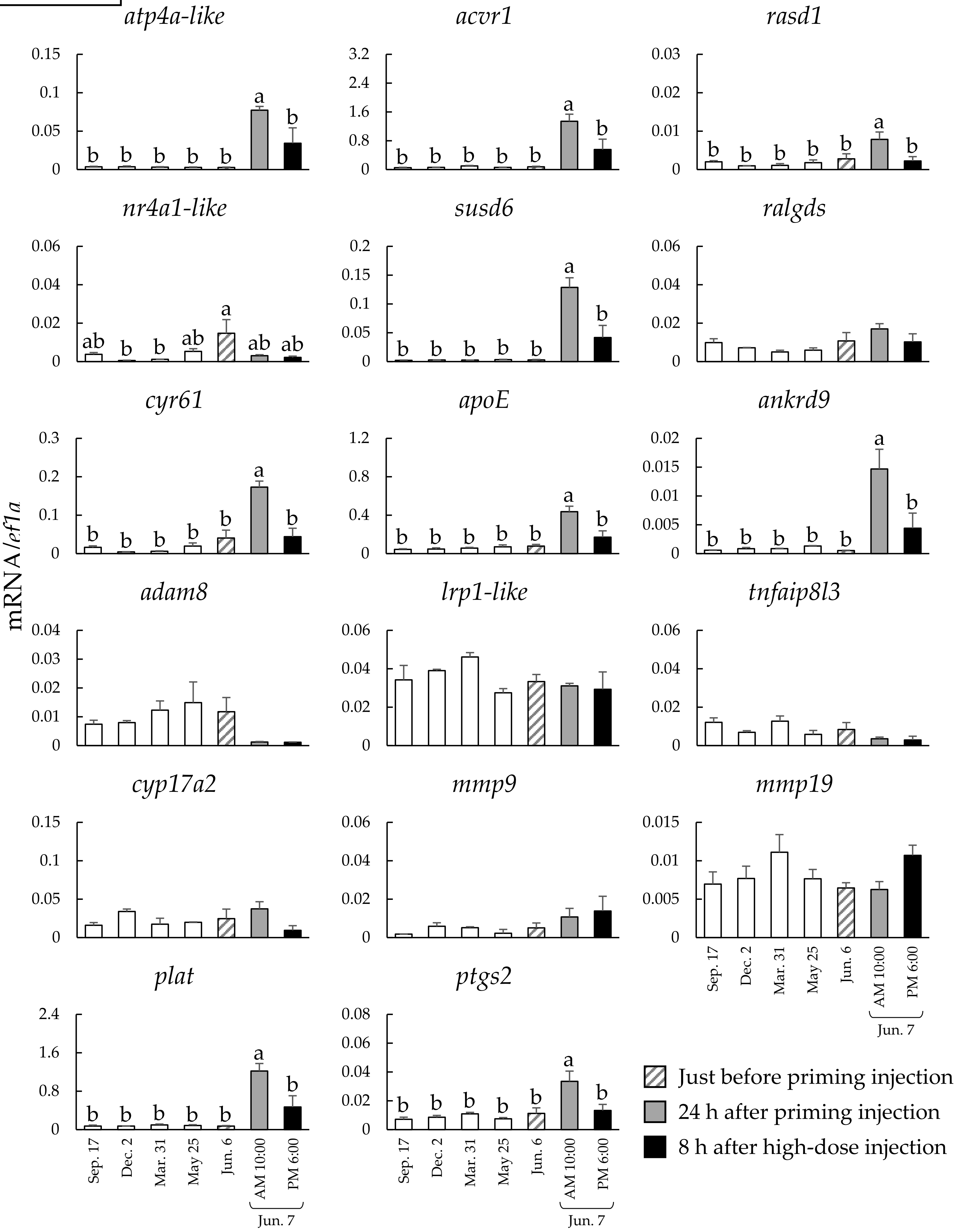

Supplemental Figure S4

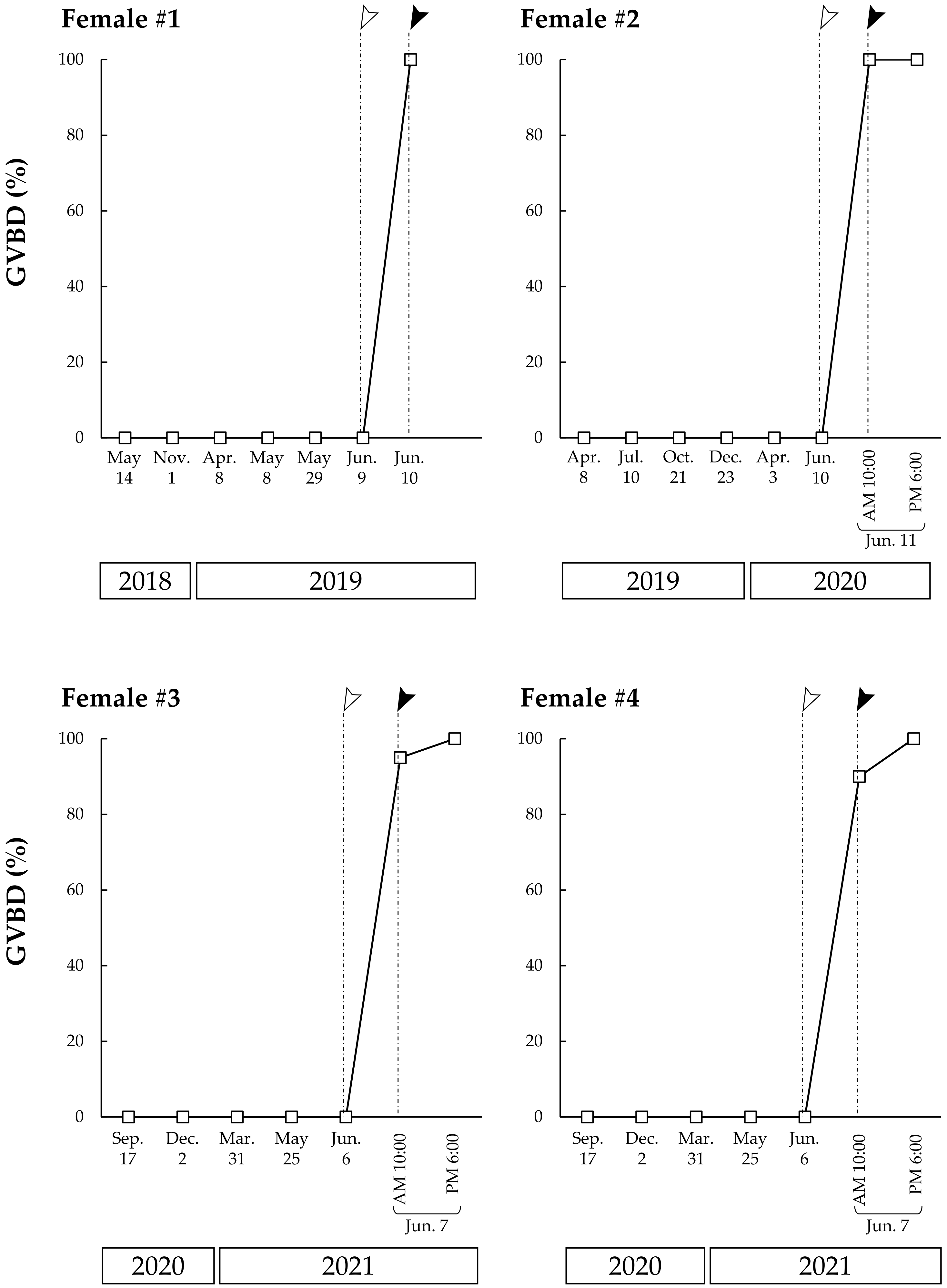

Supplemental Figure S5

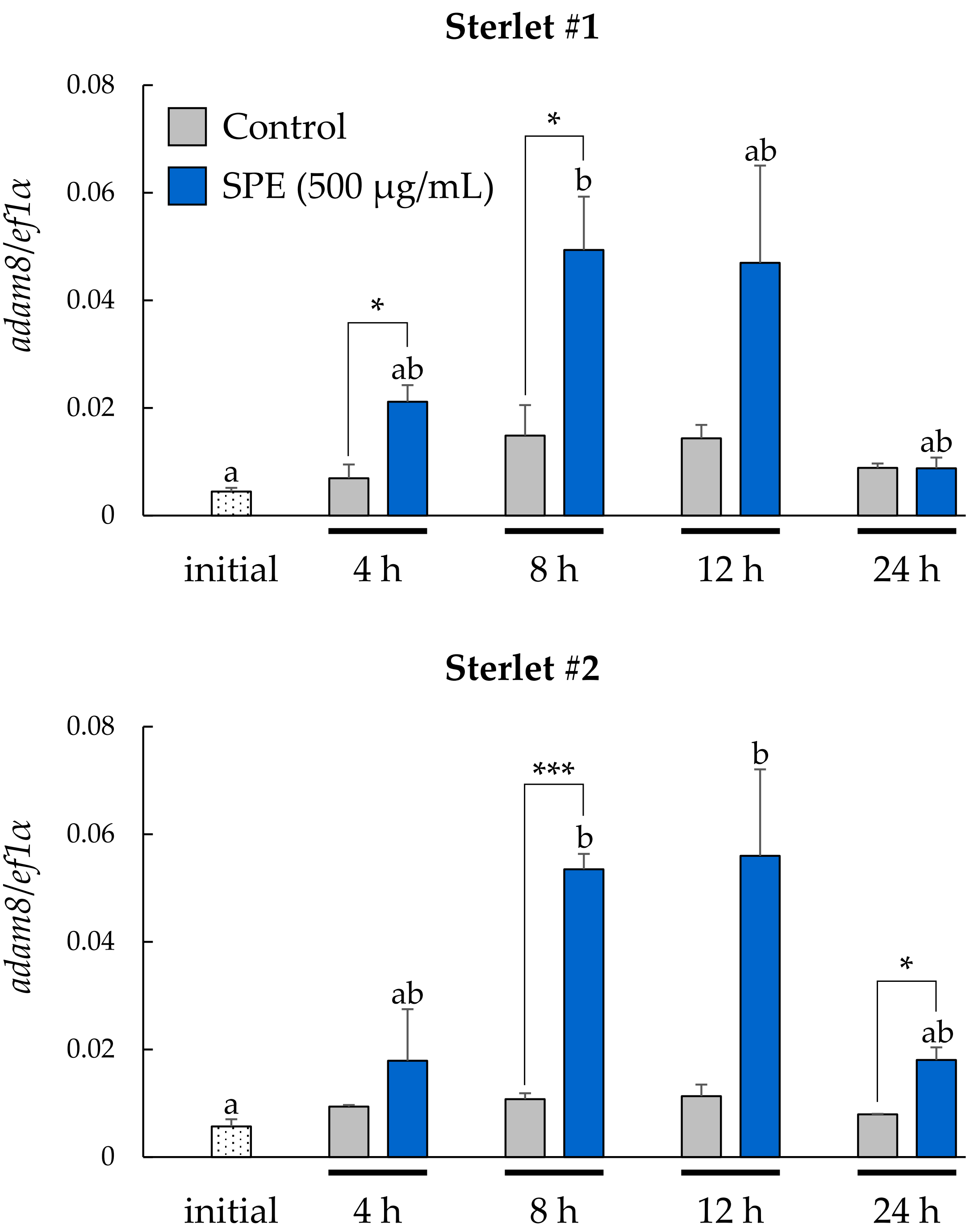

Supplemental Figure S6

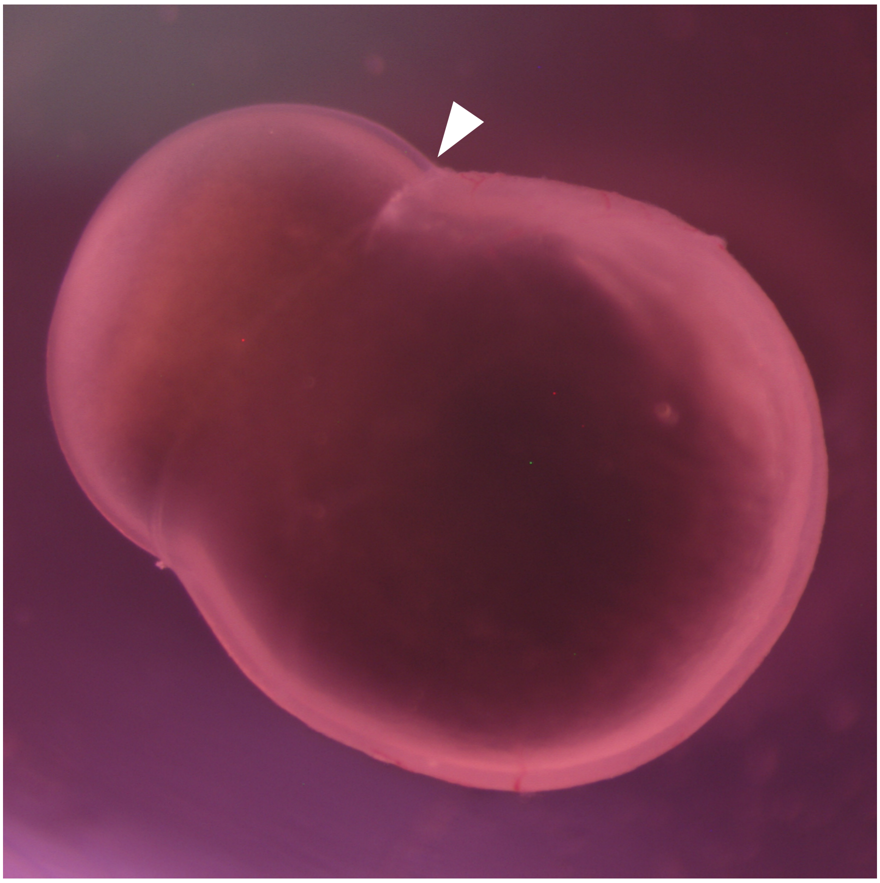

Supplemental Figure S7
